# Isolation and Characterization of Primary Retinal Microglia From the Human Post-mortem Eyes for Future Studies of Ocular Diseases

**DOI:** 10.3389/fncel.2021.786020

**Published:** 2022-01-13

**Authors:** Luoziyi Wang, Yiwen Qian, Xin Che, Jing Jiang, Jinshan Suo, Zhiliang Wang

**Affiliations:** Department of Ophthalmology, Huashan Hospital, Fudan University, Shanghai, China

**Keywords:** human post-mortem eye, retina, microglia, primary cell culture, immune response

## Abstract

Microglia, the primary resident immunocytes in the retina, continuously function as immune system supervisors in sustaining intraocular homeostasis. Microglia relate to many diseases, such as diabetic retinopathy, glaucoma, and optic nerve injury. To further investigate their morphology and functions *in vitro*, a reliable culture procedure of primary human retinal microglia is necessary. However, the culture condition of microglia from the adult retina is unclear. Researchers created several protocols, but most of them were carried out on rodents and newborns. This study describes a protocol to isolate and characterize human primary retinal microglia from human post-mortem eyes. The whole procedure started with removing the retinal vessels, mechanical separation and enzymatic dissociation, filtration, and centrifugation. Then, we cultured the cell suspensions on a T-75 flask for 18 days and then shook retinal microglia from other retinal cells. We found numerous retinal microglia grow and attach to Müller cells 10 days after seeding and increase rapidly on days 14–18. Iba1 and P2RY12 were used to qualify microglia through immunofluorescence. Moreover, CD11b and P2RY12 were positive in flow cytometry, which helps to discriminate microglia from other cells and macrophages. We also observed a robust response of retinal microglia in lipopolysaccharide (LPS) treatment with proinflammatory cytokines. In conclusion, this study provides an effective way to isolate and culture retinal microglia from adult human eyes, which may be critical for future functional investigations.

## Introduction

Microglia, the primary resident immunocytes of the central nervous system (CNS), continuously function as immune system supervisors in sustaining intracerebral homeostasis and modulating physiological and pathological processes (Ginhoux et al., 2010; Kettenmann et al., 2011). They originated from primitive hematopoietic progenitors in the extraembryonic yolk sac during embryonic and post-natal development (Schulz et al., 2012; Ginhoux and Prinz, 2015). Resembling microglia in the CNS, microglia in the retina have similar morphologies and functions. They play an essential role in tissue development, infectious response, and damage repair. Earlier reports showed that retinal microglia might come from two main sources outside the retinal parenchyma during the developing stage. One is the blood vessels of the ciliary body and iris, and the other is the retinal vasculature (Diaz-Araya et al., 1995). Usually, retinal microglia are found in the ganglion cell layer (GCL), inner and outer plexiform layers, and around the vessels with different morphologies (Diaz-Araya et al., 1995; Chen et al., 2002; Reichenbach and Bringmann, 2020). They keep retinal homeostasis *via* regulating intra-retinal cell contacts and cytokine secretions from neurons and retinal pigment epithelium (Langmann, 2007). Besides, due to their close relationship with blood-derived immunocytes and macroglia, retinal microglia can initiate inflammatory responses to defend microorganisms and lead to tissue injuries by releasing proinflammatory cytokines, reactive oxygen species (ROS), and reactive nitrogen species (RNS). Retinal microglia are involved in the pathogenesis of inflammatory ocular diseases, such as diabetic retinopathy, glaucoma, and optic nerve injury. Therefore, it is essential to study human primary retinal microglia *in vitro* to understand their roles better. However, more *in vitro* studies of human primary retinal microglia are still needed to understand their roles better.

An update described the commonly used microglia cell lines, including mouse (BV2, N9), rat (HAPI), and human cell lines (HMO6, HMC3, and C13NJ), also stem cell-derived microglia (Timmerman et al., 2018). Initially, researchers contributed to finding an effective method for culturing brain microglia *in vitro*. Mechanical separation combined with enzymatic dissociation is applied in most parts of the brain tissue, followed by a density gradient centrifugation. Meanwhile, researchers also performed similar protocols on rodents, even pigs, mostly newborns and neonatal, to explore the characteristics and functions of retinal microglia *in vitro*. However, the cultural condition of human retina microglia has still not reached a consensus so far (Wang et al., 2007; Ma et al., 2009; Devarajan et al., 2014; Lim et al., 2019).

In this study, we isolated adult human microglia from post-mortem eyeballs and investigated their characteristics. We found that microglia are in the GCL (plexiform layers), which were consistent with previous findings (Chen et al., 2002). A simple method was established by combining mechanical separation and enzymatic dissociation. After isolated, cell suspensions were resuspended in DMEM-F12 media until shaken from mixed cells on day 14. Immunofluorescence and flow cytometry were used for further characterization. Lipopolysaccharide (LPS) significantly induced the upregulation of proinflammatory cytokines related to the M1 macrophage.

## Materials and Methods

### Eyeball Tissue

Samples in this article were eyeball residues of adult human donors (*n* = 5) in Huashan Hospital, Fudan University. These eyeballs were acquired following the tenets of the Declaration of Helsinki for Research Involving Human Tissue. In brief, all the donors had previously signed the notarized agreements to dedicate their cornea for medical use and were screened serologically negative for viral infection pre-operatively. Furthermore, all the blood relatives of the donors have given informed consent in detail about the procedure of ophthalmectomy and the probability that the donated tissues could be used for research, and then, they agreed on written consent approved by the Moral and Ethical Committee of Huashan Hospital, Fudan University to allow enucleation performed on dead. Typically, the eyeballs were applied for kerectomy right after the enucleation. Then, the eye tissue leftovers were harvested after dissection of the corneal graft and preserved at 4°C in DMEM-F12 media (containing 1% penicillin, 1% streptomycin, and 1% amphotericin B) for less than 2 h. The subsequent isolating operations were performed within 6 h.

### Dissection of the Eye

After corneal transplantation, the residual eyeballs still maintained their original shapes. We used autoclaved ophthalmic forceps and scissors for dissection. The eyeballs were washed twice by pre-cooled phosphate buffered saline (PBS) in a 10 cm dry dish and carefully transferred into another dish with a pre-cooled DMEM-F12 medium. An initial circular incision posterior to the cornea limbus was made, and then, all anterior segments, including iris ciliary bodies, lens, and vitreous body, were carefully removed. The posterior eyecup left was dissected into 4 quadrants, and then, the sensory retina was gently removed and placed in a dish containing a washing medium. All vessels were carefully stripped from the retina without disturbing the retina pigment epithelium (RPE) layer due to the firm attachment, especially at the optic disc. Then, the retina was washed thoroughly to ensure that no RPE remained.

### Retinal Microglia Isolation

Cut the residual retinal tissues into pieces of approximately 1 mm^2^ by forceps and scissors, and then, dissociated the pieces mechanically using a 10-ml pipette until no more feeling of resistance and enzymatically using Collagenase A (0.2%, Sigma Aldrich) for 40 min. Fully digested tissue suspensions were passed through a 70-μm cell strainer, then washed by a pre-warmed DMEM-F12 medium with an equal volume of the suspensions, and centrifuged for 10 min (400 × *g*, 4°C). The supernatants were discarded, and the precipitates were resuspended for filtration and centrifugation once again. The cell pellets were washed twice and resuspended in a pre-cooled culture media (DMEM/F12), supplemented with 10% fetal bovine serum (FBS, Gibco), 1% penicillin-streptomycin (Pen-Strep, Invitrogen), 1% GlutaMAX (Gibco), 1% amphotericin B (Gibco), 1% sodium pyruvate (Gibco), and 1%MEM non-essential amino acid solution (Gibco). Cell viability was calculated with trypan blue. Harvested cells were seeded in T75 flasks at a density of 3–4 × 10^5^ cells/cm^2^ in DMEM-F12 culture media (37°C, 5% CO_2_). The culture medium was first exchanged on the third day after plating and then exchanged once a week. Ultimately, each human retina produced 15–20 million cells. On day 17, T75 flasks were shaken for 2 h on an orbital shaker at 37°C. The supernatant was collected and spun down at 400 *g* for 10 min, and cell viability was detected before cell seeding on plates for further experiments.

### Cell Morphology

During the induction stage, cellular morphology was observed daily and monitored by optical microscopy (Leica) equipped using the NIS-Elements Advanced Research software (Nikon, Japan). The exposure settings in each separate experiment were kept constant for further comparison.

### Retinal Sections

The eyeballs were fixed in 4% paraformaldehyde (PFA) for 48 h at 4°C, embedded in optimal cutting temperature compound (OCT compound) (Tissue-Tek, Naperville, IL, United States), and frozen at −20 to −80°C. Then, the eye tissue was cut into slides of 5-μm thick along the vertical meridian and transferred onto gelatin-coated slides. Finally, the slides were dried on a slide warmer for 30 min at 37°C and then stored at −20 to −80°C before using them.

### Immunocytochemistry

After isolating on the first day, a small part of mixed retinal cells was seeded at a density of 1 × 10^6^ cells per well in a 24-well plate with poly-L-lysine-coated glass coverslips at the bottom. For cultured retinal microglia, we cultured them after shaking on day 17 at a density of 2 × 10^5^ cells per well onto poly-L-lysine-coated glass coverslips in a 24-well plate. After attachment to the coverslips, cells were deprived of medium and fixed with 4% PFA for 1 h. Then, 0.1% Triton was used for permeabilization for 20 min. Then, 5% bovine serum albumin (BSA, Sigma) was performed as a blocking buffer. Primary antibodies of anti-ionized calcium-binding adaptor molecule 1 (Iba1, Abcam, #ab178846, 1:200) and anti-Purinergic Receptor P2Y12 (P2RY12, Biolegend, #848001, 1:100) were used to stain the microglia-related marker at 4°C overnight. Primary antibodies were incubated at 4°C overnight. The next day, coverslips or tissue sections were incubated with secondary antibodies (Alexa Fluor 488 of goat anti-mouse, Yeasen, 1:1,000; Alexa Fluor 647 of goat anti-rabbit, Yeasen, 1:1,000) at room temperature for 1 h. DAPI (Abcam, #ab228549, 1:1,000) was used for staining nuclear DNA. The morphology of retinal microglia and their specific locations in retina layers were observed under immunofluorescence microscopy (Leica) equipped with the NIS-Elements Advanced Research software.

### Retinal Microglia Stimulation

Isolated primary retinal microglia were seeded at a density of 2 × 10^5^ per well in 24-well plates in 500 μl medium mentioned above and treated with (LPS from *Escherichia coli* O55:B5, Sigma, #L6529, 100 ng/ml) for 24 h.

### Real-Time Polymerase Chain Reaction

After LPS stimulation, TRIzol Reagent (Life Technologies, United States) was used to extract purified RNA. The total mRNA of each sample was extracted according to the protocol of the manufacturer and eluted in RNase-free water. Quantitative polymerase chain reaction (PCR) was performed using the Applied Biosystems QuantStudio 6 Flex Real-Time PCR System.

PCR primers employed were as follows:

Glyceraldehyde-3-phosphate dehydrogenase (GAPDH) forward primer 5′-ACCCACTCCTCCACCTTTGA-3′; reverse primer 5′-CTGTTGCTGTAGCCAAATTCGT-3′ IL-6 forward primer 5′-CCAGCTATGAACTCCTTCTC-3′; reverse primer 5′-GCTTGTTCCTCACATCTCTC-3′; IL-8 forward primer 5′- ATGACTTCCAAGCTGGCCGTGGCT-3′; reverse primer 5′-TCTCAGCCCTCTTCAAAAACTTCTC-3′; MCP-1/CCL-2 forward primer 5′-CGCTCAGCCAGATGCAATCAA-3′; reverse primer 5′-GTGGTCCATGGAATCCTGAACC-3′; IL-1α forward primer 5′-CAGAAGACCTCCTGTCCTATGAGG-3′; reverse primer 5′-GCTGTGCAGAGGAACCA-3′; IL-1β forward primer 5′-AAGCTGATGGCCCTAAACAG-3′; reverse primer 5′-AGGTGCATCGTGCACATAAG-3′; TNF-α forward primer 5′-CCCAGGCAGTCAGATCATCTTC-3′; reverse primer 5′-GTGAGGAGCACATGGGTGGAG-3′; cycling conditions were conducted as described (polymerase activation at 95°C 30 s; 40 cycles, 95°C, 5 s; 60°C, 34 s). The data were analyzed using the 2^–ΔΔCt^ method.

### Flow-Cytometric Analysis

Flow cytometry evaluated the CD11b and P2RY12 positive cell fractions for proper separation of microglia from other cell types. These cells were subsequently harvested, fixed, and then analyzed by flow cytometry using anti-P2RY12 and CD11b. The labeled cells are represented by the blue-shaded populations, whereas the red populations depict the unlabeled cells. Gates were analyzed for the number and percentage of cells. For P2RY12 (PE-labeled, Biolegend) and CD11b (APC-labeled, Biolegend), appropriate isotype controls were regularly included to assess background levels of fluorescence. The viability of the cells was analyzed using the fixable viability dye 7-AAD (eBioscience, #00-6993-50). The intensity of expression on CD11b and P2RY12 was measured as mean fluorescence intensity (MFI). MFI was used to quantify the fluorescence of each observed discrete peak. Fluorescence was analyzed using FlowJo™ Software version X (BD, United States).

## Statistical Analysis

All data analyses were performed using GraphPad Prism version 9.0 software. Results were expressed as means ± SE of the mean (SEM). All data were represented as at least three independent replicates of independent experiments. Statistical analysis was performed using paired and unpaired Student’s *t*-test, and the differences between groups were compared using Tukey’s test. Probability values of *P* < 0.05 were considered to be statistically significant.

## Results

### Retinal Microglia Located in the Ganglion Cell Layer and Around the Cessels

**FIGURE 1:**
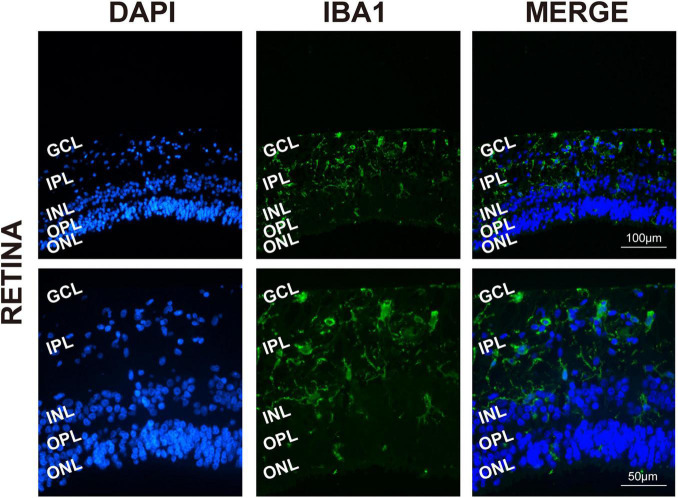
Retinal microglia located in the GCL and around the vessels. Representative immunofluorescence photomicrographs of retinal microglia marker Iba1 to label the location of retinas (GCL, ganglion cell layer; IPL, inner plexiform layer; INL, inner nuclear; ONL, outer nuclear layer). Scale bar: 100 μm.

To determine the presence and distribution of retinal microglia in the eye tissues, Iba1 and DAPI were used, respectively, for microglia labeling and nuclei staining. As expected, Iba1 immunoreactivity examination showed retinal microglia mainly located in the GCL and inner plexiform layers (IPL), presenting as a classical resting ramified morphology in Figure 1 (Chen et al., 2002).

### Morphology of Human Post-mortem Retinal Microglia

**FIGURE 2:**
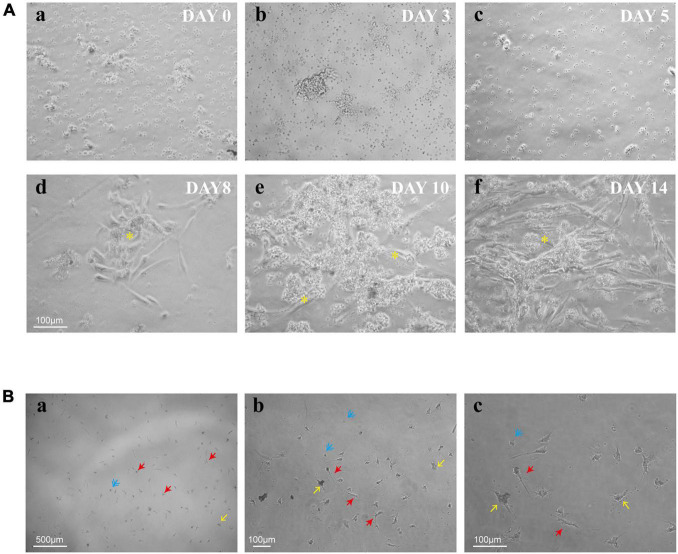
Timeline for the culture of retinal microglia. **(A)** Representative phase-contrast microscopy images of retinal mixed glial culture seeded on T75 flasks at a density of 3–4 × 10^5^ cells/cm^2^ (a). The first media change was made on day 3 after cell adherent (b). Small granules of cell debris presenting as black dots were shown up on the plate surface on day 5 (c). Cells grew and emerged to clusters (*) on day 8 and attached to Müller cells (d). Müller cells were spread all over the bottom of the plate, and retinal microglia were shown as small bright rounded cells which loosely adhered to Müller cells (e,f). **(B)** Shaken retinal microglia were shown multiple cell morphologies (blue: rounded; yellow: ameboid; and red: ramified). Scale bar: 500 μm in a; 100 μm in b; and 100 μm in c.

To explore the process of human retinal microglia from isolation to emergence, we kept on observing and photographing the dynamically living cells under light microscopy (Figure 2Aa). Adherent cells were detected on day 3 (Figure 2Ab), and the culture medium was first exchanged on that day after plating, and then once a week. Small granules of cell debris presenting as black dots were observed on the plate surface on day 5 (Figure 2Ac). After being cultured for 8 days, Müller cells presented as cylindrical, fiber-like shapes (Figure 2Ad). Meanwhile, small, bright, and rounded cells loosely adhered to Müller cells with the appearance of other differentiated retina cells, such as fibroblasts (Figure 2Ae). On day 14, Müller cells spread all over the bottom of the plate (Figure 2Af), and the loosely suspended microglia were shaken from Müller cells and replated into cell culture dishes or plates for further studies. After shaking from flasks, multiple cell morphologies were observed, including rounded, ameboid, and ramified shape of resting ramified microglia (Figure 2B).

### Retinal Microglia Highly Expressed Ionized Calcium-Binding Adaptor Molecule 1 and Purinergic Receptor P2Y12

**FIGURE 3:**
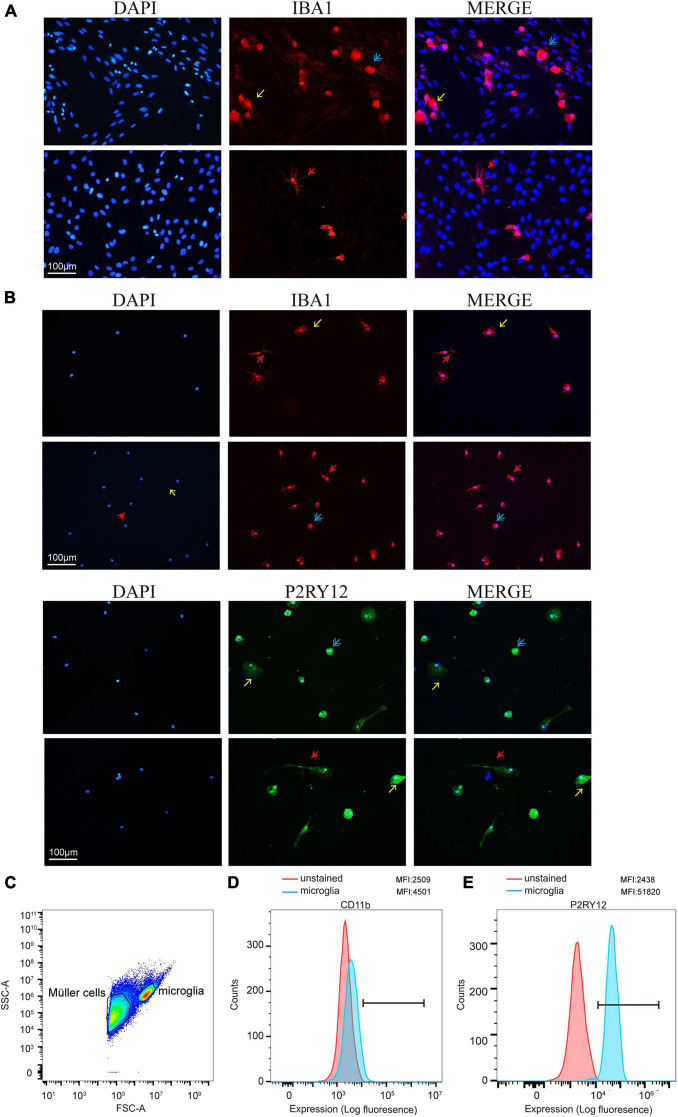
Retinal microglia expressed characteristic cell markers. **(A)** Iba1 was used to specify cultured retinal microglia before being shaken (red). **(B)** Immunofluorescence staining of cells was shown positive for microglia markers Iba1 and P2RY12 after shaking from Müller cells and plating on a 24-well plate. **(C)** FACS plot displaying the gates for both Müller cells and microglia cells. **(D,E)** Data presented show mean fluorescence intensity (MFI) of CD11b and P2RY12 expression on microglia. CD11b and P2RY12 positive cells were shown as blue populations and non-labeled as red (Blue: rounded; yellow: ameboid; red: ramified; and scale bar: 100 μm).

To determine the presence of retinal microglia among all the retina cells, we used Iba1 to specify microglia through immunofluorescence during the culture stage (Figure 3A). As a specialized macrophage, microglia have not only common biomarkers with macrophages, such as Iba1, but also unique markers, such as P2RY12, which is absent on macrophages. Still, Iba1 and P2RY12 were found significantly highly expressed retinal microglia as expected through immunofluorescence after shaking from flasks (Figure 3B). In addition, the MFI of CD11b and P2RY12 was assessed by flow cytometry, showing a significant change in labeled cells represented by the blue shaded populations, which needs further confirmation (Figures 3C–E).

### Lipopolysaccharide-Induced Retinal Microglia Exhibited Proinflammatory Reactions

**FIGURE 4:**
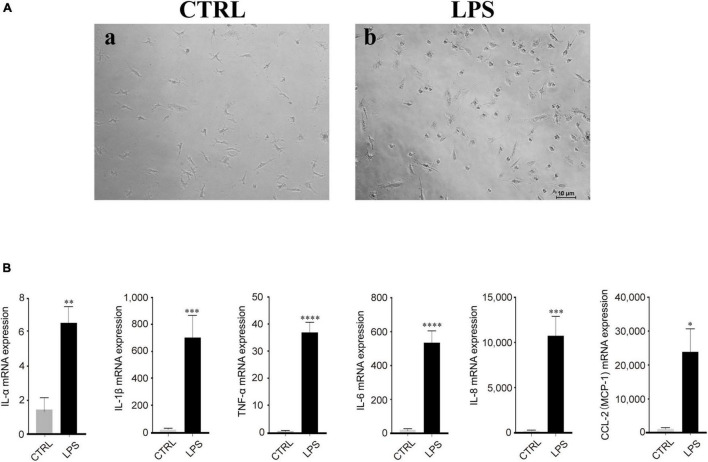
Lipopolysaccharide (LPS)-induced retinal microglia exhibited proinflammatory reactions. **(A)** Representative images of retinal microglia under 24 h LPS (100 ng/ml) stimulation manifested as morphological changes from the original polarized shape to an ameboid-like shape (Blue: rounded; yellow: ameboid; red: ramified; scale bar: 100 μm). **(B)** LPS stimulated retinal microglia showed significant mRNA expression of IL-1α/β, IL-6, IL-8, MCP-1/CCL-2, and TNF-α by qRT-PCR. Data were analyzed using an unpaired *t*-test. These assays were repeated three times. All data were normally distributed. *P < 0.05, ^**^P < 0.01, ^***^P < 0.001, and ^****^P < 0.0001.

Based on our observation, the effect of LPS treatment (100 ng/ml) manifested as morphological changes from the original polarized shape to an ameboid-like shape (Figure 4A). In addition, RT-PCR analysis showed that the mRNA expression of proinflammatory cytokines, such as IL-1α/β, IL-6, IL-8, MCP-1, CCL-2, and TNF-α, was increased after LPS stimulation, which means that these cytokines were produced by activated microglia and was strongly and significantly induced in the microglia by LPS treatment (Figure 4B).

## Discussion

Microglia have been proved an essential mediator in pathogenesis in brain and eye diseases. Over the past 100 years, the methods of isolation and culture of microglia are still unclear. Researchers have discovered many methods to identify brain microglia on mice, rats, pigs, and *post-mortem* human brain tissues and have set up yielding protocols (Giulian and Baker, 1986; Sedgwick et al., 1991; Dick et al., 1997; Mizee et al., 2017). Although the biological characteristics of microglia in the retina are similar to those in the CNS, the cellular environments of these two are different, especially in adults. Immortalized microglial cell lines, such as BV2, HAPI, and HMC3, and stem cell-derived microglia are used for studying ocular diseases *in vitro* due to their easy accessibility, but they are not appropriate for all the applications due to the different origins and immature types. Therefore, for further study of adult human retinal microglia-related ocular disease *in vitro*, it is necessary to establish a reliable technique to extract and culture primary retinal microglia. The earliest was the adherent method for selective growth and was deeply studied on rats and mice (Matsubara et al., 1999; Harada et al., 2002). Then, additional shaking steps to collect loosely adherent microglia were referred to in this study as the shaking method contrary to the selective growth of microglia in the adherent method (Wang et al., 2007; Ma et al., 2009; Dong et al., 2012). However, these previous methods are all limited to acquiring microglia on animals.

We adopted the previous method used in mice, rats, and pigs based on intraocular evidence. Our modified protocol initially removed all the anterior segments and retinal vessels without disturbing the residual retina. Then, the retinal tissues were cut into pieces and digested by Collagenase A. Previously, researchers from the University of Sydney had proposed a protocol for culturing retinal microglia. The key procedure was to digest the dissected retina with trypsin and then use 70 and 44 μm meshes to filter (Diaz et al., 1998; Pham et al., 2005).

This study illustrates a method to isolate retinal microglia from human post-mortem eye tissues by digesting previously dissociated retinas using Collagenase A, which is compared with trypsin. The digestion time was prolonged due to the gentle reaction, but the damage to tissues was relatively mild, and the digestion was efficient so that only 70 μm cell strainer was enough for filtration to harvest the retinal microglia successfully. In this study, we observed that retinal microglia adherent to the plate on the third day after being plated in DMEM/F12 media till confluence. Müller cells covered the plate and were attached by retinal microglia from day 10. On day 14, retinal microglia were shaken from Müller cells and showed different morphologies, including round, amoeboid, and ramified shapes. These retinal microglia were morphologically similar to the brains. Our data showed that retinal microglia are mainly located in the GCL and IPL, presenting as its typical ramified morphology, consistent with the previous findings.

In the next step to verify the isolated retinal microglia, the extracted cells were characterized through immunofluorescence and flow cytometric analysis. The results showed that they were expressed appropriate levels of cell surface proteins. Iba1, the microglia/macrophage-specific calcium-binding protein, helps identify target cells initially among all the retinal cells (Imai et al., 1996). But only Iba1-positive cells cannot be discriminated from other blood-derived monocytes or other tissue-resident macrophages. Furthermore, the expression of P2RY12—the unique protein of microglia—was measured and showed a high expression level in cultured cells (Butovsky et al., 2014; Lecchi et al., 2015). Moreover, the MFI of CD11b and P2RY12 were elevated, giving strong support to discriminate retinal microglia from other cells, even macrophages.

We also observed a robust response of retinal microglia in LPS treatment with the production of proinflammatory cytokines, such as IL-1α/β, IL-6, IL-8, MCP-1/CCL-2, and TNF-α, which were known as products of activated microglia. The significant upregulation of these cytokines induced by LPS treatment in retinal microglia indicated the activation of microglia into the proinflammatory (M1) state. In conclusion, our study provides a feasible method for the isolation and characterization of adult human retinal microglia from post-mortem eye tissues. It also suggests that microglia in the human retina are distributed in the GCL as ramified shape. Retinal microglia can retain their morphologies and be activated beyond LPS stimulation. The new method of this study had two limitations. The first one is the rare acquirement of human eye tissues and the limited control of sex and age. The second is the post-mortem conditions that might affect the microglial phenotype. For the quantification of microglia from initial dissociation to the end of the protocol, we found that the yield is different due to the sex and age of the owner of the eyeballs. Therefore, it is necessary to compare the effect of different conditions on yield and phenotypes of microglia, such as sex, age, cause of death, and post-mortem delay.

In summary, our finding may be constructive for establishing an efficient isolation and culture method of primary human retinal microglia. This finding is crucial for future studies about retinal microglia.

## Data Availability Statement

The raw data supporting the conclusions of this article will be made available by the authors, without undue reservation.

## Author Contributions

All authors listed have made a substantial, direct, and intellectual contribution to the work, and approved it for publication.

## Conflict of Interest

The authors declare that the research was conducted in the absence of any commercial or financial relationships that could be construed as a potential conflict of interest.

## Publisher’s Note

All claims expressed in this article are solely those of the authors and do not necessarily represent those of their affiliated organizations, or those of the publisher, the editors and the reviewers. Any product that may be evaluated in this article, or claim that may be made by its manufacturer, is not guaranteed or endorsed by the publisher.

## References

[B1] Butovsky O. Jedrychowski M. P. Moore C. S. Cialic R. Lanser A. J. Gabriely G. (2014). Identification of a unique TGF-beta-dependent molecular and functional signature in microglia. *Nat. Neurosci.* 17 131–143. 10.1038/nn.3599 24316888 PMC4066672

[B2] Chen L. Yang P. Kijlstra A. (2002). Distribution., markers., and functions of retinal microglia. *Ocul. Immunol. Inflamm.* 10 27–39. 10.1076/ocii.10.1.27.10328 12461701

[B3] Devarajan G. Chen M. Muckersie E. Xu H. (2014). Culture and characterization of microglia from the adult murine retina. *ScientificWorldJournal* 2014:894368. 10.1155/2014/894368 24987746 PMC4060747

[B4] Diaz C. M. Penfold P. L. Provis J. M. (1998). Modulation of the resistance of a human endothelial cell line by human retinal glia. *Aust. N. Z. J. Ophthalmol.* 26 S62–S64. 10.1111/j.1442-9071.1998.tb01376.x 9685026

[B5] Diaz-Araya C. M. Provis J. M. Penfold P. L. Billson F. A. (1995). Development of microglial topography in human retina. *J. Comp. Neurol.* 363 53–68. 10.1002/cne.903630106 8682937

[B6] Dick A. D. Pell M. Brew B. J. Foulcher E. Sedgwick J. D. (1997). Direct ex vivo flow cytometric analysis of human microglial cell CD4 expression: examination of central nervous system biopsy specimens from HIV-seropositive patients and patients with other neurological disease. *AIDS* 11 1699–1708. 10.1097/00002030-199714000-00006 9386804

[B7] Dong N. Li X. Xiao L. Yu W. Wang B. Chu L. (2012). Upregulation of retinal neuronal MCP-1 in the rodent model of diabetic retinopathy and its function in vitro. *Invest. Ophthalmol. Vis. Sci.* 53 7567–7575. 10.1167/iovs.12-9446 23010641

[B8] Ginhoux F. Prinz M. (2015). Origin of microglia: current concepts and past controversies. *Cold Spring Harb. Perspect. Biol.* 7:a020537. 10.1101/cshperspect.a020537 26134003 PMC4526747

[B9] Ginhoux F. Greter M. Leboeuf M. Nandi S. See P. Gokhan S. (2010). Fate mapping analysis reveals that adult microglia derive from primitive macrophages. *Science* 330 841–845. 10.1126/science.1194637 20966214 PMC3719181

[B10] Giulian D. Baker T. J. (1986). Characterization of ameboid microglia isolated from developing mammalian brain. *J. Neurosci.* 6 2163–2178. 10.1523/JNEUROSCI.06-08-02163.1986 3018187 PMC6568755

[B11] Harada T. Harada C. Kohsaka S. Wada E. Yoshida K. Ohno S. (2002). Microglia-Muller glia cell interactions control neurotrophic factor production during light-induced retinal degeneration. *J. Neurosci.* 22 9228–9236. 10.1523/JNEUROSCI.22-21-09228.2002 12417648 PMC6758038

[B12] Imai Y. Ibata I. Ito D. Ohsawa K. Kohsaka S. (1996). A novel gene iba1 in the major histocompatibility complex class III region encoding an EF hand protein expressed in a monocytic lineage. *Biochem. Biophys. Res. Commun.* 224 855–862. 10.1006/bbrc.1996.1112 8713135

[B13] Kettenmann H. Hanisch U. K. Noda M. Verkhratsky A. (2011). Physiology of microglia. *Physiol. Rev.* 91 461–553. 21527731 10.1152/physrev.00011.2010

[B14] Langmann T. (2007). Microglia activation in retinal degeneration. *J. Leukoc. Biol.* 81 1345–1351. 10.1189/jlb.0207114 17405851

[B15] Lecchi A. Razzari C. Paoletta S. Dupuis A. Nakamura L. Ohlmann P. (2015). Identification of a new dysfunctional platelet P2Y12 receptor variant associated with bleeding diathesis. *Blood* 125 1006–1013. 10.1182/blood-2013-07-517896 25428217 PMC4319231

[B16] Lim R. R. Hainsworth D. P. Mohan R. R. Chaurasia S. S. (2019). Characterization of a functionally active primary microglial cell culture from the pig retina. *Exp. Eye Res.* 185:107670. 10.1016/j.exer.2019.05.010 31103710 PMC13552233

[B17] Ma W. Zhao L. Fontainhas A. M. Fariss R. N. Wong W. T. (2009). Microglia in the mouse retina alter the structure and function of retinal pigmented epithelial cells: a potential cellular interaction relevant to AMD. *PLoS One* 4:e7945. 10.1371/journal.pone.0007945 19936204 PMC2775955

[B18] Matsubara T. Pararajasegaram G. Wu G. S. Rao N. A. (1999). Retinal microglia differentially express phenotypic markers of antigen-presenting cells in vitro. *Invest. Ophthalmol. Vis. Sci.* 40 3186–3193. 10586941

[B19] Mizee M. R. Miedema S. S. van der Poel M. Adelia, Schuurman K. G. van Strien M. E. (2017). Isolation of primary microglia from the human post-mortem brain: effects of ante- and post-mortem variables. *Acta Neuropathol. Commun.* 5:16. 10.1186/s40478-017-0418-8 28212663 PMC5316206

[B20] Pham V. T. Wen L. McCluskey P. Madigan M. C. Penfold P. L. (2005). Human retinal microglia express candidate receptors for HIV-1 infection. *Br. J. Ophthalmol.* 89 753–757. 10.1136/bjo.2004.057828 15923514 PMC1772690

[B21] Reichenbach A. Bringmann A. (2020). Glia of the human retina. *Glia* 68 768–796. 10.1002/glia.23727 31793693

[B22] Schulz C. Gomez Perdiguero E. Chorro L. Szabo-Rogers H. Cagnard N. Kierdorf K. (2012). A lineage of myeloid cells independent of Myb and hematopoietic stem cells. *Science* 336 86–90. 10.1126/science.1219179 22442384

[B23] Sedgwick J. D. Schwender S. Imrich H. Dorries R. Butcher G. W. ter Meulen V. (1991). Isolation and direct characterization of resident microglial cells from the normal and inflamed central nervous system. *Proc. Natl. Acad. Sci. U S A* 88 7438–7442. 10.1073/pnas.88.16.7438 1651506 PMC52311

[B24] Timmerman R. Burm S. M. Bajramovic J. J. (2018). An overview of in vitro methods to study microglia. *Front. Cell Neurosci.* 12:242. 10.3389/fncel.2018.00242 30127723 PMC6087748

[B25] Wang A. L. Yu A. C. He Q. H. Zhu X. Tso M. O. (2007). AGEs mediated expression and secretion of TNF alpha in rat retinal microglia. *Exp. Eye Res.* 84 905–913. 10.1016/j.exer.2007.01.011 17359975

